# Targeted Drug-Loaded Chemical Probe Staining Assay to Predict Therapy Response and Function as an Independent Pathological Marker

**DOI:** 10.1016/j.isci.2019.10.050

**Published:** 2019-10-28

**Authors:** Heng Zhang, Wei-long Zhong, Bo Sun, Guang Yang, Yan-rong Liu, Bi-jiao Zhou, Xin Chen, Xiang-yan Jing, Long-cong Huai, Ning Liu, Zhi-yuan Zhang, Mi-mi Li, Jing-xia Han, Kai-liang Qiao, Jing Meng, Hong-gang Zhou, Shuang Chen, Cheng Yang, Tao Sun

**Affiliations:** 1 State Key Laboratory of Medicinal Chemical Biology and College of Pharmacy, Nankai University, Tianjin, China; 2 Tianjin Key Laboratory of Early Druggability Evaluation of Innovative Drugs and Tianjin Key Laboratory of Molecular Drug Research, Tianjin International Joint Academy of Biomedicine, Tianjin, China; 3 Department of Gastroenterology and Hepatology, Tianjin Medical University General Hospital, Tianjin Institute of Digestive Disease, Tianjin, China

**Keywords:** Drugs, Medical Biochemistry, Biochemical Assay

## Abstract

Multi-targeted kinase inhibitors, such as sorafenib, have been used in various malignancies, but their efficacy in clinical applications varies among individuals and lacks pretherapeutic prediction measures. We applied the concept of “click chemistry” to pathological staining and established a drug-loaded probe staining assay. We stained the cells and different types of pathological sections and demonstrated that the assay was reliable. We further verified in cells, cell-derived xenograft model, and clinical level that the staining intensity of the probe could reflect drug sensitivity. The stained samples from 300 patients who suffered from hepatocellular carcinoma and used the sorafenib probe also indicated that staining intensity was closely related to clinical information and could be used as an independent marker without undergoing sorafenib therapy for prognosis. This assay provided new ideas for multi-target drug clinical trials, pre-medication prediction, and pathological research.

## Highlights

• The assay could be used for the rapid sensitivity detection of multi-target drugs
• The assay could serve as an auxiliary technology for NGS and PDX
• Sorafenib had multiple unknown primary contribution targets
• Drug-probe staining could be used as an independent diagnostic and prognostic marker

## Graphical Abstract

**Figure:**
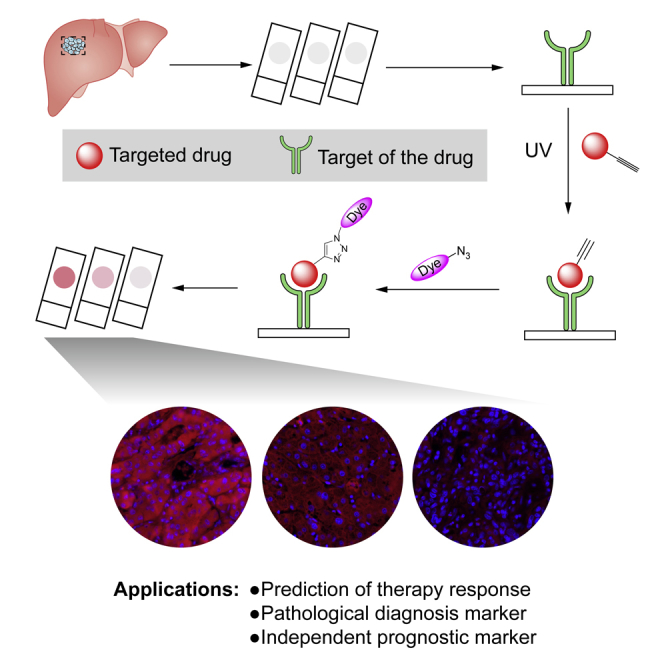

## Introduction

Targeted drug therapy is regarded as the main treatment for malignant tumors. Next-generation sequencing (NGS) has been widely used as a clinical diagnostic method for targeted drugs (Khotskaya et al., 2017, Prasad et al., 2016). In lung cancer and breast cancer treatments, tyrosine-kinase inhibitors, a kind of single-target drug, can accurately target a certain kinase or its mutation (Grimminger et al., 2010). A class of targeted drugs exists, and it includes multi-target kinase inhibitors, such as sorafenib (Wilhelm et al., 2006), which is the first drug approved for the treatment of advanced hepatocellular carcinoma (HCC) (Raoul et al., 2018). This class of drugs cannot rely on NGS for premedication diagnosis. Accurately predicting a target's level in clinical trials during drug development is also difficult (Llovet et al., 2008). In clinical applications, the therapeutic effects of multi-target drugs often vary among individuals, and their therapeutic effects are unstable and random, which are similar to traditional chemotherapy (Garraway and Hahn, 2010). Consequently, they become a problem in the clinical use of targeted drugs. The efficacy and adaptation of a population can be confirmed through large multi-center clinical trials or meta-analysis. For instance, sorafenib can inhibit up to 40 kinases, including angiogenic receptor tyrosine kinases (RTKs), such as VEGF receptors (VEGFRs) and PDGF receptor-β (PDGFRβ), and play a role in anti-angiogenesis and antitumor proliferation (Wilhelm et al., 2008). Biomarkers capable of predicting sorafenib reactivity have yet to be discovered because of the diversity and pharmacological complexity of sorafenib targets. Although patient-derived tumor xenografts (PDTX) can be used to predict drug sensitivity, this method is time consuming, costly, and difficult to use universally. NGS is also difficult to predict the therapy response of these drugs (Tentler et al., 2012). Therefore, efficient methods should be developed to predict whether a patient can respond to multi-target drugs and guide clinical use (Llovet et al., 2018).

On the basis of our previous work (Zhong et al., 2016), we introduced a pathological staining assay by using a targeted drug-loaded probe based on chemical probes used for drug target research. The assay could evaluate the subcellular location and relative expression of a drug target in a surgical resection tissue or a biopsy specimen in a short period and predict the reaction of patients to a drug. The assay was independent of NGS and could be compared with pathological immunohistochemistry and H&E staining for an effective coordination.

The term “click chemistry” was first fully described by Sharpless in 2001 (Kolb et al., 2001) and has been widely used in biochemical labeling (Meghani et al., 2017, Wright and Sieber, 2016). The classic click reaction is the copper-catalyzed reaction of an azide with an alkyne to form a five-membered heteroatom ring (Rostovtsev et al., 2002). In this study, we modified the inactive functional group of the original drug molecule with a terminal alkyne as a drug probe to allow minimal functionalization. We added the probe to the test sample, which bound to the target protein, and linked azide-tagged rhodamine to the probe *in vitro* via a click reaction, or a copper-catalyzed azide–alkyne cycloaddition reaction, which enabled fluorescence to represent the characteristics of the drugs. To reduce the probability of a probe off-target and increase the binding force and sensitivity of the probe and the drug, we introduced the light affinity group (double acridine) to the probe under ultraviolet (UV) exposure. The group could combine with the amino acid near the drug pocket to form covalent binding so that the probe and the target protein bind more closely (Li and Zhang, 2016).

To test the feasibility of this assay, we used a classic target drug, namely, single-target imatinib, to establish the probe staining assay combined with IF of its target CD117 and other methods and evaluate the reliability of the proposed method on gastrointestinal stromal tumor (GIST) (Joensuu et al., 2013). Our results showed that the assay worked. We also designed the multi-target drug sorafenib probe and applied it to predict drug reactivity (sensitivity) in HCC and confirm targets. Probe staining result suggested that sorafenib staining positive cluster could be used as an independent prognostic indicator for pathological diagnosis.

## Results

### Imatinib Probe Could Bind to CD117

**Figure 1:**
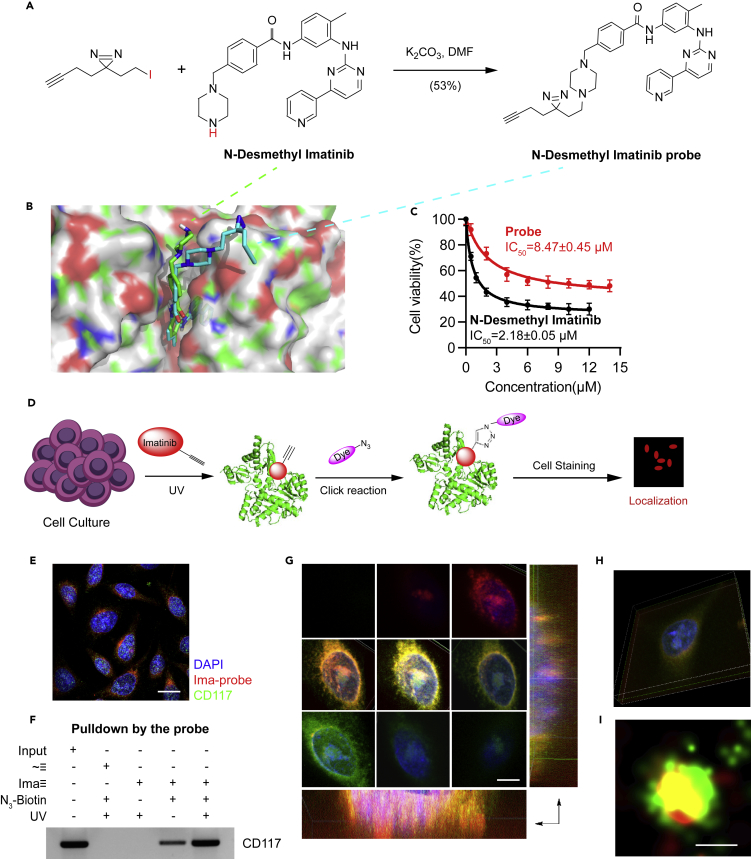
Imatinib Probe Could Bind to CD117 (A) Chemical synthesis route of imatinib probe. (B) Imatinib and probes were docked with CD117 protein. (C) Effect of imatinib and probe on proliferation of GIST882 cell line. Each bar represents the mean ± SD for triplicate experiments. (D) Schematic diagram of cell staining with imatinib probe. (E) Simultaneous staining with imatinib probe and CD117 immunofluorescence staining on the GIST882 cell line, scale bar = 10 μm. (F) Target was captured on the GIST882 cell line using N_3_-biotin and probe in combination with pulldown and detected using Western blot analysis of CD117. (G) Imatinib probe staining and CD117 immunofluorescence staining, z axis sweep with confocal microscopy, scale bar = 5 μm. (H) Three-dimensional reconstruction of Figure 1G after sweeping. (I) Co-localization of imatinib probe staining and CD117 immunofluorescence in single molecule levels observed using a super resolution microscope, scale bar = 0.1 μm.

On the basis of the structure–activity relationship of imatinib, we determined that the probe-modified position was a nonpocket-binding functional base (Manley et al., 2010). Therefore, the synthetic route shown in Figure 1A was designed, the imatinib probe was obtained, and the structure was confirmed through nuclear magnetic resonance (NMR) (Figure S1). The probe was studied in terms of its ability to bind well to a target because its structure differed from that of the original drug. We first evaluated whether the activity of the probe was similar to that of the original drug. Using the computer docking program, we docked imatinib (green) and the probe (blue) with their target CD117. In Figure 1B, the conformation of the two combined with the CD117 active pocket was similar, and the probe-modified group was on the outer side of the active pocket. We further used surface plasmon resonance (SPR) to investigate the binding affinity of imatinib and the probe to CD117. As shown in Figure S2, binding of imatinib and the probe to CD117 was dose dependent, exhibiting a fast association-dissociation process. The response units at equilibrium were plotted against imatinib and the probe concentrations, and the dissociation constant (KD) was calculated by non-linear regression, suggesting that the binding affinity of imatinib and the probe to CD117 was similar. Conducting the CCK-8 assay, we tested the effect of imatinib and its probe on the proliferation of the imatinib-sensitive gastrointestinal stromal tumor cell line GIST882. In Figure 1C, the curves of imatinib and imatinib-probe were similar in shape with IC_50_ of 2.18 and 8.47 μM, indicating that the activity of the two cells had the same order of magnitude. Next, we stained the cells with the imatinib probe by applying the procedure shown in Figure 1D and observed the colocalization of the CD117 fluorescence (Figure 1E). We also used confocal three-dimensional layer sweep (Figures 1G) and 3D reconstruction (Figure 1H) to demonstrate that probe staining was colocalized with CD117 on the membrane. Super-resolution microscopy revealed that they combined well with high specificity in a single molecule level (Figure 1I). Pearson correlation coefficient (PCC) was 0.744, and Mander's overlap coefficient (MOC) was 0.759. We also used the imatinib probe pulldown to confirm that CD117 was detected through Western blot analysis (Figure 1F). These results indicated that the probe could bind to the target of the original drug and produce a similar inhibitory activity.

### Establishment of Pathological Section Staining by Using the Imatinib Probe

**Figure 2:**
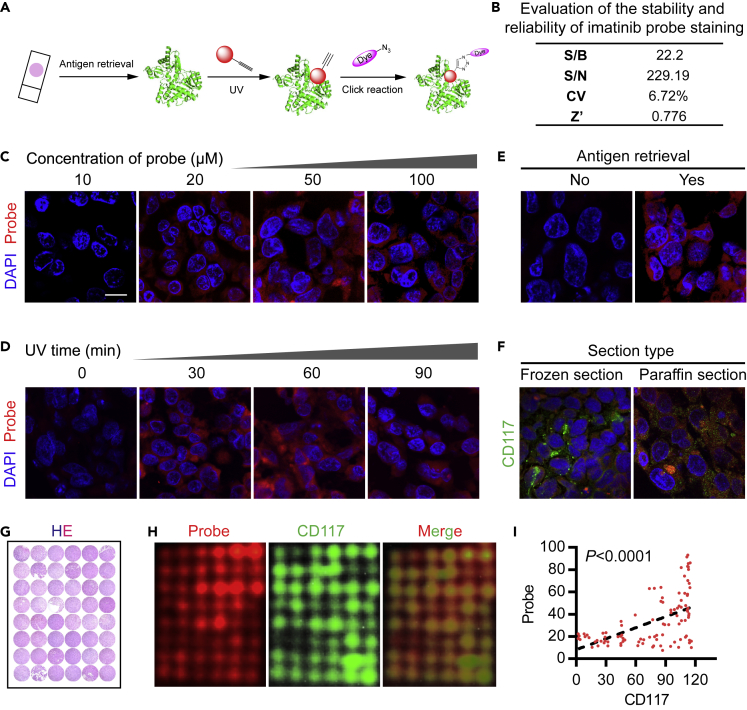
Establishment of Pathological Section Staining Process Using Imatinib Probe (A) Procedure for staining the imatinib probe on tissue sections. (B) Evaluation of imatinib probe stability and reliability. (C–E) Staining results obtained under different conditions: the concentration of the probe (C), the time of UV exposure (D), and antigen retrieval (E), scale bar = 10 μm. (F) Imatinib probe staining and CD117 immunofluorescence staining on frozen sections and paraffin sections. (G) HE staining of the GIST tissue microarray. (H) Staining with the imatinib probe (red) and CD117 immunofluorescence staining (green) on the GIST tissue microarray. (I) Correlation analysis of probe staining and CD117 staining on the GIST tissue microarray.

We stained the pathological sections in accordance with the procedure shown in Figure 2A and evaluated the stability and reliability of staining (Figure 2B). The Z factor of staining was 0.776, and the coefficient of variation (CV) was 6.72%. We explored the following conditions of the key steps: the concentration of the probe (Figure 2C), the time of UV exposure (Figure 2D), and antigen retrieval (Figure 2E). We found that microwave antigen retrieval could be achieved in the paraffin sections of the conventional formalin-fixed specimens at a probe concentration of 50 μM and a UV exposure duration of 60 min. On the basis of these experimental conditions, we compared the staining effects on frozen sections and paraffin sections. The comparison with CD117 immunofluorescence showed that they had fine colocalization (Figure 2F). The frozen sections could be stained without antigen retrieval, and the paraffin sections were more suitable than the frozen sections for antigen retrieval. On the basis of these findings, we collected 24 pathological paraffin specimens of gastrointestinal stromal tumors from well-diagnosed patients in clinical and fabricated tissue microarrays (HE staining image shown in Figure 2G) to meet the fluorescence analysis requirement under the same conditions. Probe staining and immunofluorescence on the tissue chip were similar to those on the cells and had a fine colocalization effect (Figure 2H); MOC was 0.904 and PCC was 0.711. The staining intensity of the probe was significantly correlated with the CD117 fluorescence intensity (p < 0.0001; Figure 2I), suggesting that the probe staining assay was effective.

### Preparation of Sorafenib Probe for Fluorescence Staining

**Figure 3:**
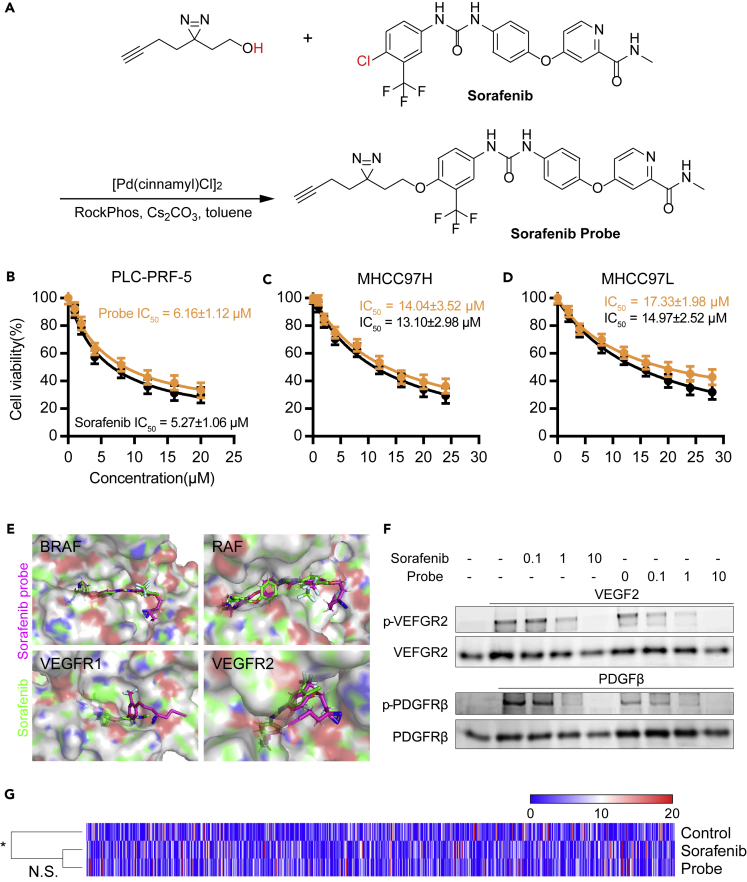
Preparation of Sorafenib Probe for Fluorescence Staining (A) Chemical synthesis route of sorafenib probe. (B–D) Effects of different concentrations of sorafenib and probe on the proliferation of PLC-PRF-5 (B), MHCC97H (C), and MHCC97L (D) cell lines. Each bar represents the mean ± SD for triplicate experiments. (E) Sorafenib and probes were mock docked with four known targets of sorafenib. (F) Effects of different concentrations of sorafenib and probe on the expression of VEGFR2, PDGFRβ, and phosphorylation at the target. (G) Expression profiles of the PLC-PRF-5 cell lines treated with solvent, sorafenib, and probe are not significantly different between the sorafenib and probe groups, and the solvent group was significantly different from the two other groups.

After confirming the feasibility of the staining method with the single-target drug imatinib probe, we synthesized the sorafenib probe by following the synthetic route shown in Figure 3A to study if the previously described assay could solve targeted drug-related problems to some extent. On the basis of the structure–activity relationship of imatinib, According to the structure–activity relationship of sorafenib, we determined that the probe-modified position was not the binding functional base (Ramurthy et al., 2008). The probe was briefly described using several steps of synthesis, and its structure was characterized using NMR (Figure S3). We first evaluated whether the activity of the probe was similar to the activity of the original drug by applying the methods that we used for the imatinib probe. Using the CCK-8 assay, we tested the effects of sorafenib and the probe on the proliferation of HCC cell-lines PLC-PRF-5 (Figure 3B), MHCC97H (Figure 3C), and MHCC97L (Figure 3D). The results showed that sorafenib and the probe had similar IC_50_ in the three cell lines, indicating that both had similar effects on cell proliferation. Sorafenib (green) and the probe (purple) were docked with the known targets BRAF, RAF, VEGFR1, and VEGFR2 by using the computer docking program. In Figure 3E, the conformation of the binding of sorafenib and the probe to the active pockets of these targets was similar, and the terminal alkyne portion of the probe was located outside the active pocket. As shown in Figure S4, binding of sorafenib and the probe to VEGFR1 and VEGFR2 was dose dependent, exhibiting a fast association-dissociation process. The response units at equilibrium were plotted against sorafenib and the probe concentrations, and the dissociation constant (KD) was calculated by non-linear regression, suggesting that the binding affinity of sorafenib and the probe to VEGFR1 and VEGFR2 was similar. We performed Western blot analysis to detect the phosphorylation and expression of VEGFR2 and PDGFR-β in the PLC-PRF-5 cell line. The results showed that VEGFR2 phosphorylation in sorafenib and sorafenib probe treatment groups were dose-dependently reduced (Figure 3F), whereas the total amount did not change significantly. PDGFR-β phosphorylation was dose-dependently reduced, and the total amount was not significantly changed. These results indicated that the inhibitory effects of the original drug and the probe on the activity of the two targets were similar. We also used an expression profiling chip to detect the expression profile of PLC-PRF-5 cells after they were treated with sorafenib and its probe (Figure 3G). The effects of the two substances on cell expression profiles were similar, and the clustering results were not significant. The drug and probe groups were categorized into one class, and the solvent was classified into another one. These results suggested that the binding target of the sorafenib probe was consistent with that of sorafenib.

### Sorafenib Probe Staining Intensity Was Related to Drug Sensitivity

**Figure 4:**
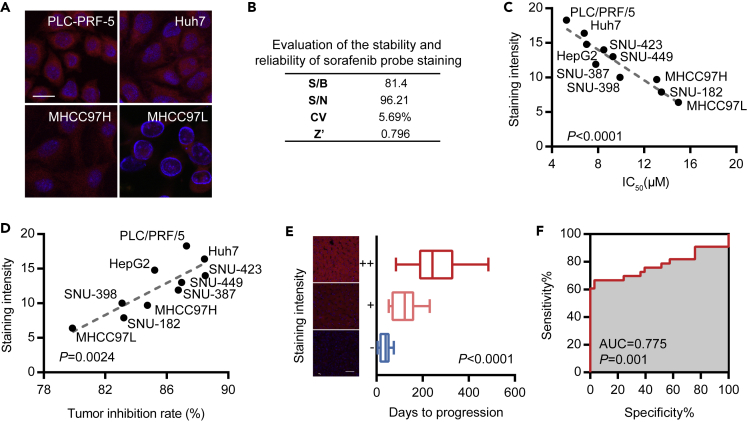
Sorafenib Probe Staining Intensity Was Related to Drug Sensitivity (A) Sorafenib probe was used to stain PLC-PRF-5, Huh7, MHCC97H, and MHCC97L cell lines; red = probe, blue = DAPI, scale bar = 15 μm. (B) Evaluation of sorafenib probe stability and reliability. (C) The staining intensity of 10 cell lines and the regression analysis of sorafenib IC_50_ showed a negative correlation. (D) After establishing cell-derived xenograft model of 10 cell lines, a positive correlation was found between tumor inhibition rate and staining intensity after treatment with sorafenib. (E) The 33 patients given with sorafenib after surgery were stained with the probe, and the PFS of strongly positive, weakly positive, and negative groups (representative results on the left, scale bar = 50 μm) was successively decreased. (F) ROC curve of sorafenib probe's prediction of sorafenib sensitivity in 33 patients.

We selected 10 cell lines and stained them with sorafenib probe. The images of PLC-PRF-5, Huh7, MHCC97H, and MHCC97L staining are presented in Figure 4A. The stability and reliability of probe staining were evaluated using the factors shown in Figure 4B. IC_50_ of sorafenib was also tested with CCK-8 in these cell lines. The results showed that sorafenib probe staining intensity was negatively correlated with IC_50_ (p < 0.0001; Figure 4C), indicating that probe staining represented drug sensitivity. We then used these 10 cell lines to establish cell-derived xenograft models and study their sensitivity to sorafenib treatment. Consistent with the cell experiment, the models demonstrated that the tumor inhibition rate of sorafenib was positively correlated with staining intensity in different cell lines (Figure 4D). We collected pathological sections of 34 patients (Table S3) who suffered from HCC and underwent sorafenib therapy after surgery and prospectively analyzed the relationship between probe staining intensity and drug efficacy. In Figure 4E, PFS increased in the three groups of patients with weak, medium, and strong probe staining (p < 0.0001), indicating that patients with strong staining were more sensitive to sorafenib treatment. On the basis of the sensitivity of the 34 patients to sorafenib as predicted by the sorafenib probe, we plotted the receiver operating characteristic (ROC) curve (Figure 4F) with an area under the curve (AUC) of 0.775, indicating that the probe staining intensity was a good predictor of drug sensitivity. These results suggested that sorafenib probe staining could reflect the drug sensitivity of sorafenib in cells, cell xenografts, and clinical levels and that probe staining could be used on surgical specimens to predict postoperative drug use.

### Meta-Analysis Showed Sorafenib Had Different Effects on Various Populations

Considering that probe staining could effectively predict the efficacy of a drug, we further determined whether probe staining could also be used for the prognostic evaluation of clinical cases or whether the effect of a drug on prognosis could be understood by slice staining before treatment to enrich the detection range of pathological diagnosis. We used the sorafenib probe to establish a predictive assay independent of treatment and targets. First, we tested whether the drug treatment of sorafenib could affect survival time to verify whether the drug was useful. Numerous clinical multicenter studies on sorafenib have been performed, but no conclusive evidence-based medical certificate has been provided. As such, we used the flow chart shown in Figure S5A for meta-analysis. We then retrieved and included five articles, and the basic information is shown in Table S4. Meta-analysis indicated that the overall survival [OS] of patients treated with sorafenib increased (hazard ratio [HR] = 0.69, 95% confidence interval [CI] 0.57–0.82; Figure S5B) compared with that of patients administered with placebo, and various effects were observed in different case groups (Table S5). The effects of sorafenib treatment on patients with extrahepatic metastasis and hepatitis B infection were not evident. In other subgroups, such as ECOG PS, vascular invasion, and hepatitis C infection groups, sorafenib exhibited significant efficacy (p < 0.05). Sorafenib also improved OS (Figure S5C) compared with that of other targeted drugs (HR = 0.88, 95% CI 0.81–0.95) and elicited various effects on different case groups (Table S6). In comparison with other targeted drugs, sorafenib significantly improved the OS of Asian populations and patients with hepatitis C infection (p < 0.05).

### Sorafenib Targets Were Complex and Closely Related to the Prognosis of HCC

**Figure 5:**
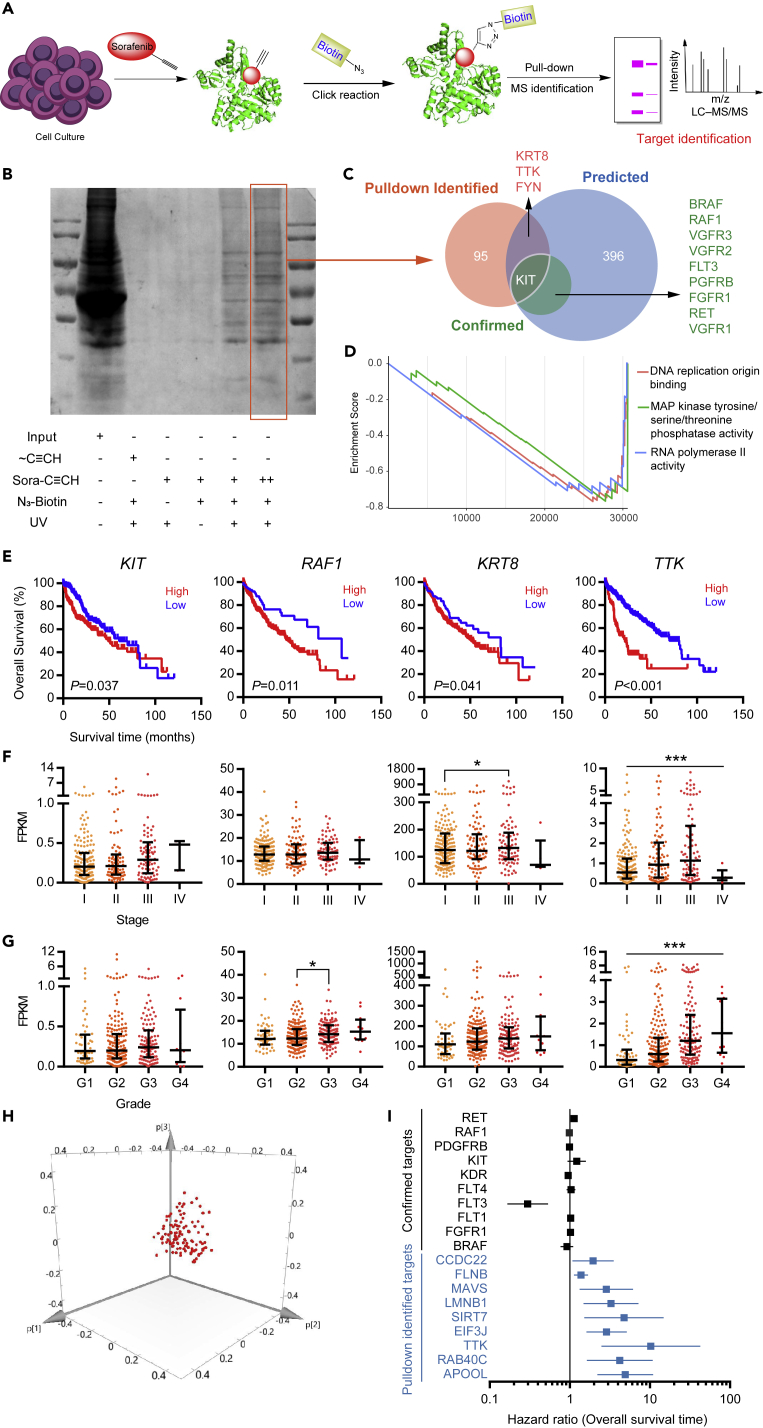
Sorafenib Targets Were Complex and Closely Related to the Prognosis of HCC (A) Schematic of fishing targets with the sorafenib probe. (B) Coomassie blue staining of the proteins after pulldown with the sorafenib probe. (C) Venn diagram of sorafenib-known targets, online predicted targets, and pulldown targets. (D) GSEA analysis after treatment of the Huh7 cell line with sorafenib. (E–G) TCGA data showed that sorafenib's known targets, namely, KIT, RAF1, fishing target KRT8, and TTK are related to HCC overall survival (E), stage (F), and grade (G). ∗p < 0.05, ∗∗∗p < 0.001. (H) PCA analysis of known and potential targets of sorafenib. (I) COX regression survival analysis of known target and fishing target of sorafenib.

The improvement of the survival of patients with HCC and the various effects of sorafenib on different cases indicated that the target group was closely related to the prognosis of HCC, and the overall expression of these target groups differed among patients. We used the probe to capture the sorafenib target (Figure 5A). The gel was run, and Coomassie blue staining (Figure 5B), enzymatic digestion, and identification via bio-mass spectrometry were performed. We comprehensively analyzed the target results obtained via pulldown, the sorafenib target contained in the Drugbank database (Wishart et al., 2018), and the prediction results obtained using online target prediction tools, namely, SEA (Keiser et al., 2007), SwissTargetPrediction (Gfeller et al., 2014) and BATMAN (Liu et al., 2016). The three sets of targets were shown in Figure 5C; many targets have not been reported, for example, TTK, KRT8, and CCDC22. The GSEA analysis after Huh7 cell line was treated with sorafenib (Won et al., 2017) validated the rationality of the obtained potential targets (Figure 5D). We analyzed some potential targets, KIT, RAF1, KRT8, and TTK by using HCC data in TCGA (Figures 5E–5G). The results showed that the high expression levels of KIT, RAF1, KRT8, and TTK were indicative of poor prognosis of HCC and were related to grade and stage. Some target expression levels had no significant relationship with the prognosis, grade, and stage of HCC (Figure S6). Sorafenib targets were complicated, but they were related to the prognosis of HCC as a whole function. Thus, the target group could be regarded as a cluster. We performed PCA on these targets by using TCGA RNA-seq data (Figure 5H) and showed that the expression of these targets was clustered. COX regression analysis revealed that the reported targets slightly contributed to prognosis, whereas the potential targets TTK, CCDC22, and SIRT7 had a high risk of prognosis of HCC (Figure 5I). Thus, the real target of sorafenib should be further studied.

### Sorafenib Target Cluster Stained Using the Sorafenib Probe Could Be Used as an Independent Prognostic Marker of HCC

Meta-analysis indicated that sorafenib treatment could improve the survival time of patients, especially in the absence of extrahepatic metastasis, vascular invasion, and other remarkable indicators. We also used biochemical methods to identify sorafenib targets, and many of these targets affect prognosis and clinicopathological parameters. Therefore, we aimed to establish a sorafenib probe stain as a novel and independent pathological stain marker for clinical prognosis evaluation. Pathological tissues stained with a sorafenib-loaded probe were not obtained from patients who took sorafenib. The staining results indicated the total expression level of sorafenib targets. We defined the expression of this total target group as “sorafenib probe cluster”, which was unrelated to the intake or non-intake of sorafenib.

**Figure 6:**
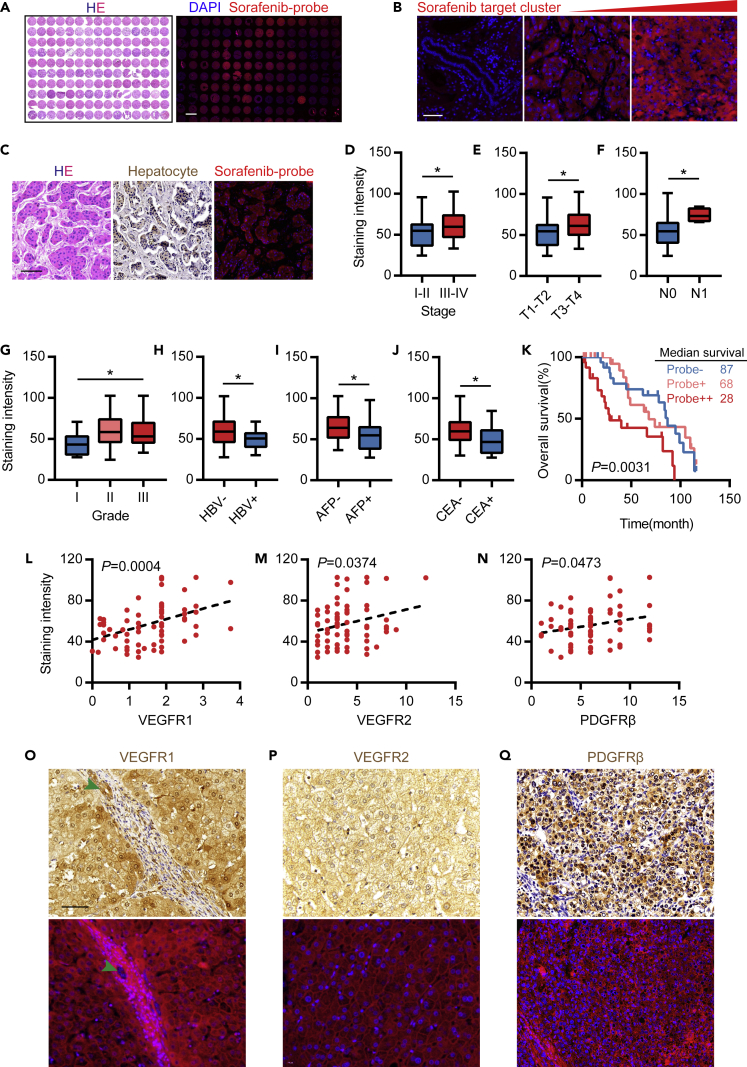
Sorafenib Target Cluster Stained with Sorafenib Probe Could Be Used as an Independent Prognosis Marker for HCC (A) HCC tissue chip HE staining and sorafenib probe staining results, scale bar = 2 cm. (B) High magnification image of sorafenib probe staining (negative, weakly positive, and strong positive), scale bar = 50 μm. (C) HE staining, hepatocyte staining, and probe staining of the same tissue. (D–J) Relationship between staining intensity and clinical stage (D), T stage (E), N stage (F), grade (G), HBV state (H), AFP (I), and CEA level (J). ∗p < 0.05. (K) KM survival analysis of strong positive, weak positive, and negative groups according to the intensity of staining. (L–N) Correlation analysis of sorafenib probe staining intensity with the three targets, namely, VEGFR1 (L), VEGFR2 (M), and PDGFRβ (N) immunohistochemical scores. (O) VEGFR1 immunohistochemical (top) and probe staining (bottom) results for the same sample with the green arrow indicating bile duct, scale bar = 50 μm. (P) VEGFR2 immunohistochemistry (top) and probe staining (bottom) in the same sample. (Q) PDGFRβ immunohistochemical (top) and probe staining (bottom) results for the same sample.

We collected 75 cases of HCC specimens with complete clinical information (Table S7) and prepared a tissue microarray. The specimens were collected from patients who did not take sorafenib and used for retrospective clinicopathological studies on probe fluorescent staining. The results of tissue microarray HE staining and fluorescence staining are shown in Figure 6A. High-magnification images of different staining intensities are shown in Figures 6B and S8. The HE staining, hepatocyte staining, and probe staining results of the same sample are illustrated in Figure 6C, indicating that the probe can be accurately positioned in the parenchyma rather than in the stroma. We analyzed staining intensity and clinical information and demonstrated that staining intensity was not significantly different in terms of gender and age (Figure S7) but was positively correlated with clinical stage (Figure 6D, p = 0.0388), T stage (Figure 6E, p = 0.0227), N stage (Figure 6F, p = 0.0369), and grade (Figure 6G, p = 0.0476). The staining results were negatively correlated with HBV status (Figure 6H, p = 0.0483), AFP (Figure 6I, p = 0.0376), and CEA level (Figure 6J, p = 0.0387). The KM survival analysis of staining intensity (Figure 6K) showed that the survival of the patients with strong positive staining was significantly shorter than that of the patients with weak positive and negative staining. The median survival periods of the three groups were 28, 68, and 87 days (p = 0.0031). We also performed immunohistochemical staining for the three sorafenib targets, namely, VEGFR1, VEGFR2, and PDGFRβ, by using the tissue microarray of the same sample and conducted correlation analysis between staining intensity and immunohistochemical score. The results showed that the staining intensity was positively correlated with the immunohistochemical scores of the three targets. Pearson correlation coefficients were 0.4158 (Figure 6L), 0.2512 (Figure 6M), and 0.2432 (Figure 6N) (p < 0.05). Representative images are shown in Figures 6O–6Q. We combined the immunohistochemical results of VEGFR1 and VEGFR2 with the sorafenib probe staining results of HCC and adjacent tissues and found that the sorafenib probe stained the blood vessels (Figure S8F green arrow; Figure S8G arrow) but did not stain the bile duct (Figure 6O green arrow and Figure S8F yellow arrow).

Sorafenib probe staining could be used not only to diagnose tumor cells but also to label stroma and reactive vessels. These results indicated that sorafenib probe staining corresponded to the expression level of total targets of sorafenib with poor prognosis and was associated with clinical stage and pathological grade. Reactive stroma and vascular invasion are markers of the poor prognosis of HCC and the main target tissue of sorafenib. The results of probe staining were consistent with the results of drug efficacy meta-analysis. Therefore, sorafenib probe cluster staining could be used as an independent prognostic marker and was independent of sorafenib treatment.

## Discussion

Drug-loaded chemical probe staining assays on pathological sections have a completely different principle from traditional chemical staining and immunohistochemical staining in pathology and are an important addition to classical pathological diagnostic techniques. In this study, the proposed assay applies click chemistry in which a fluorophore is introduced into a drug probe after it binds to a target protein to achieve fluorescent staining. As such, this assay may become an important component of immunohistochemical staining in pathology and promote the use of small-molecule drugs for pathological section staining. For example, the results of sorafenib probe staining could predict its efficacy and address NGS defects on multi-targeted drugs. The results of probe staining were also closely related to clinical pathological information and could be used as an independent pathological marker. This finding suggested that small-molecule drugs could also be transformed into probes that could be used by pathologists to study various pathological phenomena and observe the population expression levels of multiple targets.

When we used the sorafenib probe to study its target and staining, we found that its known target slightly contributed to the prognosis of HCC, but many potential targets had a high risk ratio for HCC prognosis. Thus, we proposed the concept of “drug target cluster,” which referred to total targets of a drug. Drug target cluster can be visualized by the drug-loaded probe staining assay. For a targeted drug, drug target cluster was fixed, and the staining intensity of different patient samples could be obtained through the assay. The expression level of a patient's drug target cluster could be summarized as follows: the stronger the staining, the higher the total target expression of the drug and better the expected therapeutic effect of the drug would be. Conversely, the weaker the staining, the lesser the total target expression of the drug would be in the corresponding part of the patient. Thus, the worse outcome of the effect of the targeted drug treatment was expected. This finding might help doctors determine a patient's drug sensitivity to a certain extent, that is, whether a drug was suitable for targeted drug treatment. NGS is currently recognized as a probable therapeutic predictor for single-targeted drugs and applicable to immunotherapy, but NGS is almost useless for multi-targeted drugs. Evaluating currently unknown drug targets using NGS is also impossible. Many single-target drugs also have multiple sub-targets, which often lead to missed drug use opportunities for sensitive patients. At the same time, if an epigenetic change occurs in the target of a single-target drug, this drug cannot combine with it. Although NGS shows the target is positive in a patient, the drug often appears to be ineffective. Therefore, NGS use is uncertain even for single-target drugs. The staining assay proposed in this paper could effectively assist NGS for predicting the effect of drug treatments.

Probe staining can be used not only as a predictive marker for treatment but also as an independent prognostic marker. Targeted drugs work because “drug target cluster” has a poor prognosis as a whole. After the targets are inhibited by the drug, the survival time of a patient can be improved. This observation also supports the clinical feasibility of sorafenib. Drug probe staining can indicate some pathological phenomena, including labeled reactive stroma in tumors, invaded vessels in tumors, and presence of staining signals in the nucleus. Further research is needed to determine why drug target cluster is positively associated with these pathological phenomena. Besides, active small molecules can be used not only as drugs but also as independent clinical pathological markers for specific mechanism research and clinical diagnosis. This assay would become a new type of chemical staining technology named “drug binding histochemical staining (DHC).”

When we established this staining assay, we optimized many conditions. For instance, in pathological section staining, we suggested adding a negative control and a positive control to the sample to rule out fluctuations in staining conditions, in order to reduce the probability of false negative or false positive. For this reason, we used pathological tissue microarrays to evaluate imatinib probe staining with target colocalization and the relationship of the staining intensity of sorafenib probe to clinical information to ensure that the staining conditions were identical, and the results were comparable.

A drug-loaded probe staining assay is established to reflect the drug sensitivity of sorafenib in cells, animals, and humans. Sorafenib probe staining is also associated with clinical information and pathological phenomena and thus considered an independent pathological marker. This finding may help solve the clinical problem of targeted drug sensitivity prediction and provide new ideas for the pathological diagnosis of HCC and other pathological phenomena.

### Limitations of the Study

The probe adds a terminal alkyne group and a photoaffinity group to the original drug molecule. Despite this relatively slight modification, the effect is hardly the same as the original drug, and a small portion of the probe may be combined with a nontarget protein. More sophisticated probes that are similar to the original drug structure should be further designed to improve staining specificity. This idea should also be applied to develop more probes for targeted drugs and verify the reliability and universality of the assay.

## Methods

All methods can be found in the accompanying Transparent Methods supplemental file.

## Data and Code Availability

The accession number for the data reported in this paper is GEO: GSE123600.

## Supplemental Information

Document S1. Transparent Methods, Figures S1–S9, and Tables S1–S7
