# Supplementary material for: Targeted Drug-Loaded Chemical Probe Staining Assay to Predict Therapy Response and Function as an Independent Pathological Marker
Source: iScience. 2019 Oct 28;21:549–61. doi: 10.1016/j.isci.2019.10.050 (PMC6849364; doi:10.1016/j.isci.2019.10.050)
Supplement: Document S1. Transparent Methods, Figures S1–S9, and Tables S1–S7 [file mmc1.pdf]

ISCI, Volume *21*

Supplemental Information

Targeted Drug-Loaded Chemical Probe Staining Assay to Predict Therapy Response and Function as an Independent Pathological Marker

Heng Zhang, Wei-long Zhong, Bo Sun, Guang Yang, Yan-rong Liu, Bi-jiao Zhou, Xin Chen, Xiang-yan Jing, Long-cong Huai, Ning Liu, Zhi-yuan Zhang, Mi-mi Li, Jing-xia Han, Kai-liang Qiao, Jing Meng, Hong-gang Zhou, Shuang Chen, Cheng Yang, and Tao Sun

Figure S1. The ^{1}H and ^{13}C NMR spectra of N-Desmethyl imatinib probe

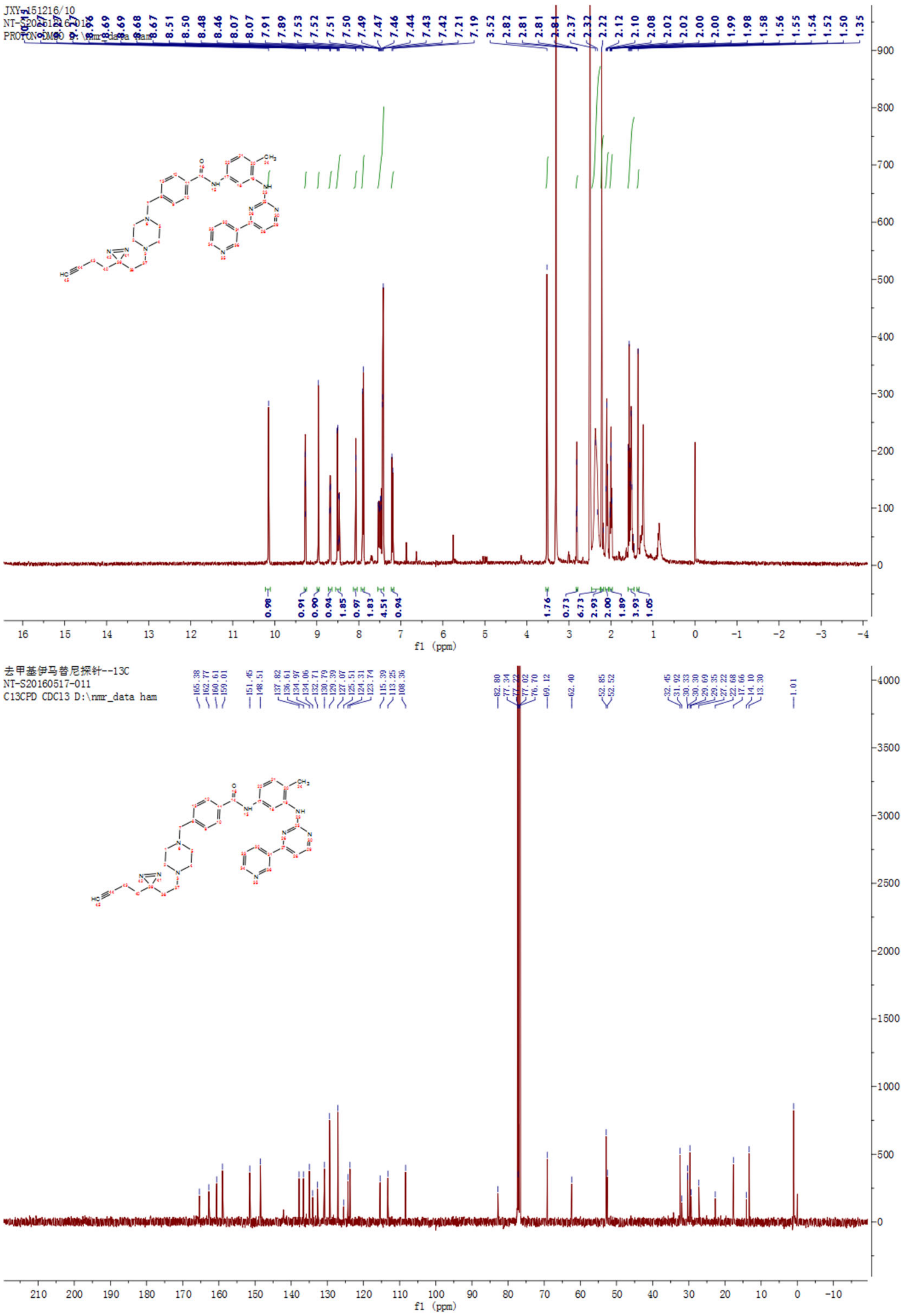

^{1}H NMR (400 MHz, DMSO) δ ppm: 10.15 (s, 1H), 9.30 – 9.24 (m, 1H), 8.96 (s, 1H), 8.68 (dd, J = 4.8, 1.6 Hz, 1H), 8.53 – 8.45 (m, 2H), 8.07 (d, J = 2.1 Hz, 1H), 7.90 (d, J = 7.9 Hz, 2H), 7.57 – 7.39 (m, 5H), 7.20 (d, J = 8.3 Hz, 1H), 3.52 (s, 2H), 2.81 (t, J = 2.7 Hz, 1H), 2.35 (d, J = 17.3 Hz, 7H), 2.22 (s, 3H), 2.10 (t, J = 7.4 Hz, 2H), 2.00 (td, J = 7.4, 2.6 Hz, 2H), 1.54 (dt, J = 17.9, 7.4 Hz, 4H), 1.35 (s, 1H).

^{13}C NMR (101 MHz, $CDCl_3$) δ ppm: 165.38, 162.77, 160.61, 159.01, 151.45, 148.51, 137.82, 136.61, 134.97, 134.06, 132.71, 130.79, 129.39, 127.07, 125.51, 124.31, 123.74, 115.39, 113.25, 108.36, 82.80, 69.12, 62.40, 52.85, 52.52, 32.45, 31.92, 30.33, 29.69, 29.35, 27.22, 22.68, 17.66, 14.10, 13.30.

ESI-MS (m/z): 600.56 ([M+H+]).

Figure S2. The binding affinity of imatinib and imatinib probe to CD117 determined using SPR

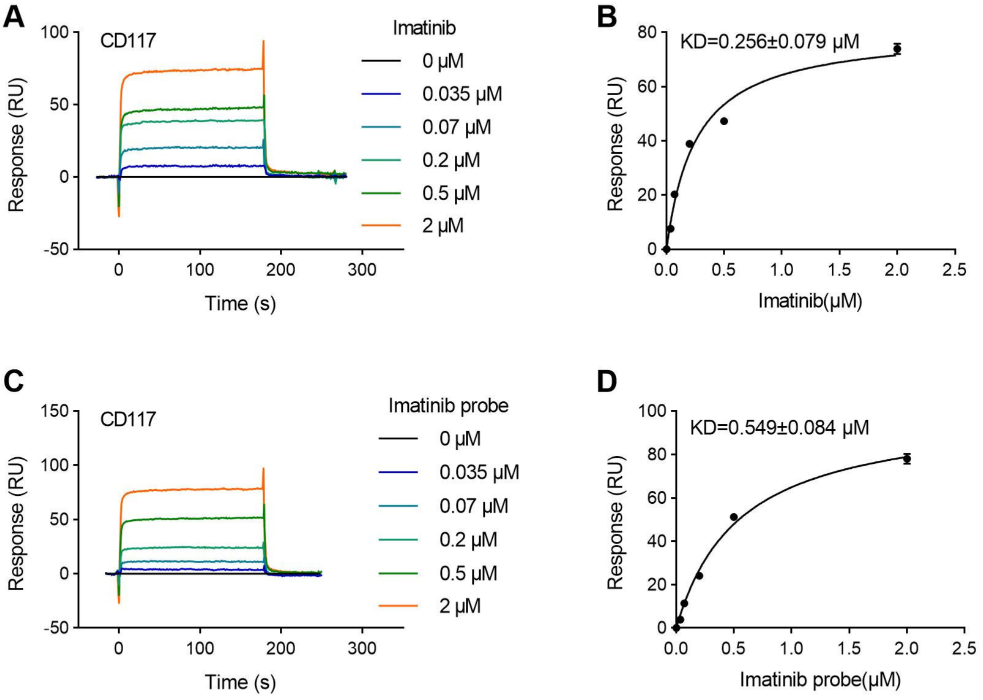

Table S1. Data used to evaluate the stability and reliability of N-Desmethyl imatinib probe staining

Fluorescence intensity	Sample	Background
Sample 1	23.780	0.850
Sample 2	23.570	1.060
Sample 3	20.620	0.880
Sample 4	24.160	0.880
Sample 5	23.670	0.990
Mean(μ)	21.360	0.962
Standard deviation(σ)	1.437	0.089

Figure S3. The ^{1}H NMR spectra of sorafenib probe

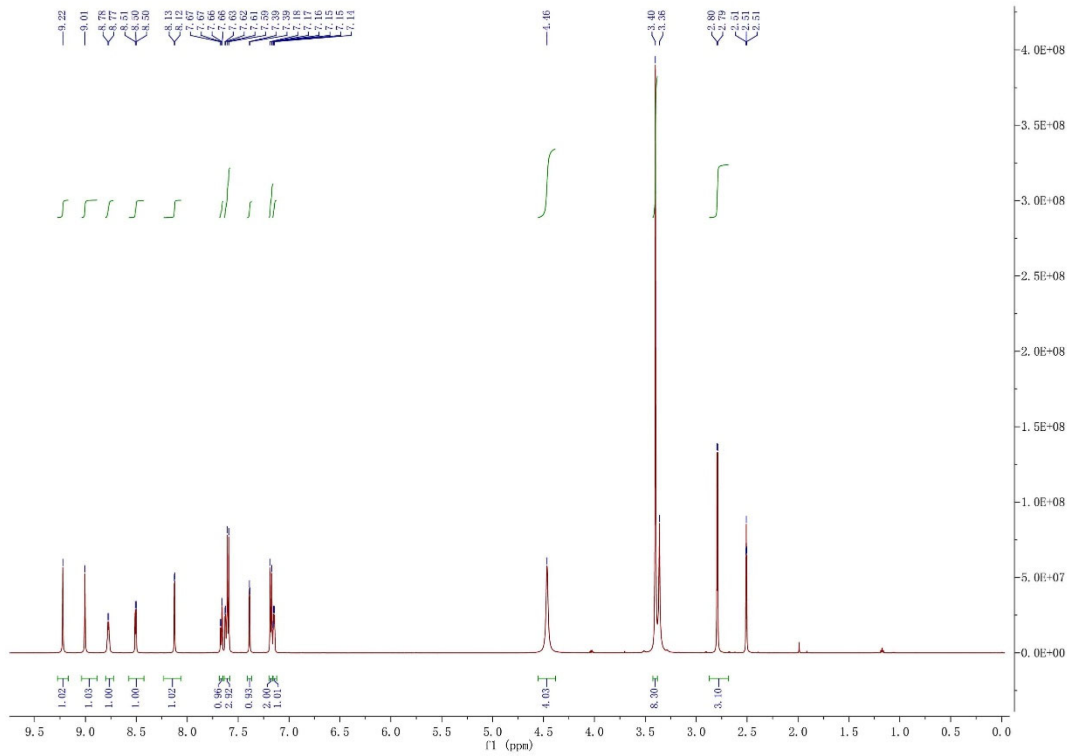

^{1}H NMR (400 MHz, $DMSO_{d6}$): δ 9.22 (s, 1H), 9.01 (s, 1H), 8.78 (d, J = 4.0 Hz, 1H), 8.50 (d, J = 4.0 Hz, 1H), 8.13 (s, 1H), 7.67 (m, 1H), 7.60 (m, J = 8.4 Hz, 2H), 7.39 (s, 1H), 7.18 (d, J = 8.4 Hz, 1H), 7.14(d, J = 4.0 Hz, 1H), 4.50 (br, 4H), 3.40 (s, 8H), 2.80 (s, 3H).

Figure S4. The binding affinity of sorafenib and sorafenib probe to VEGFR1 and VEGFR2 determined using SPR

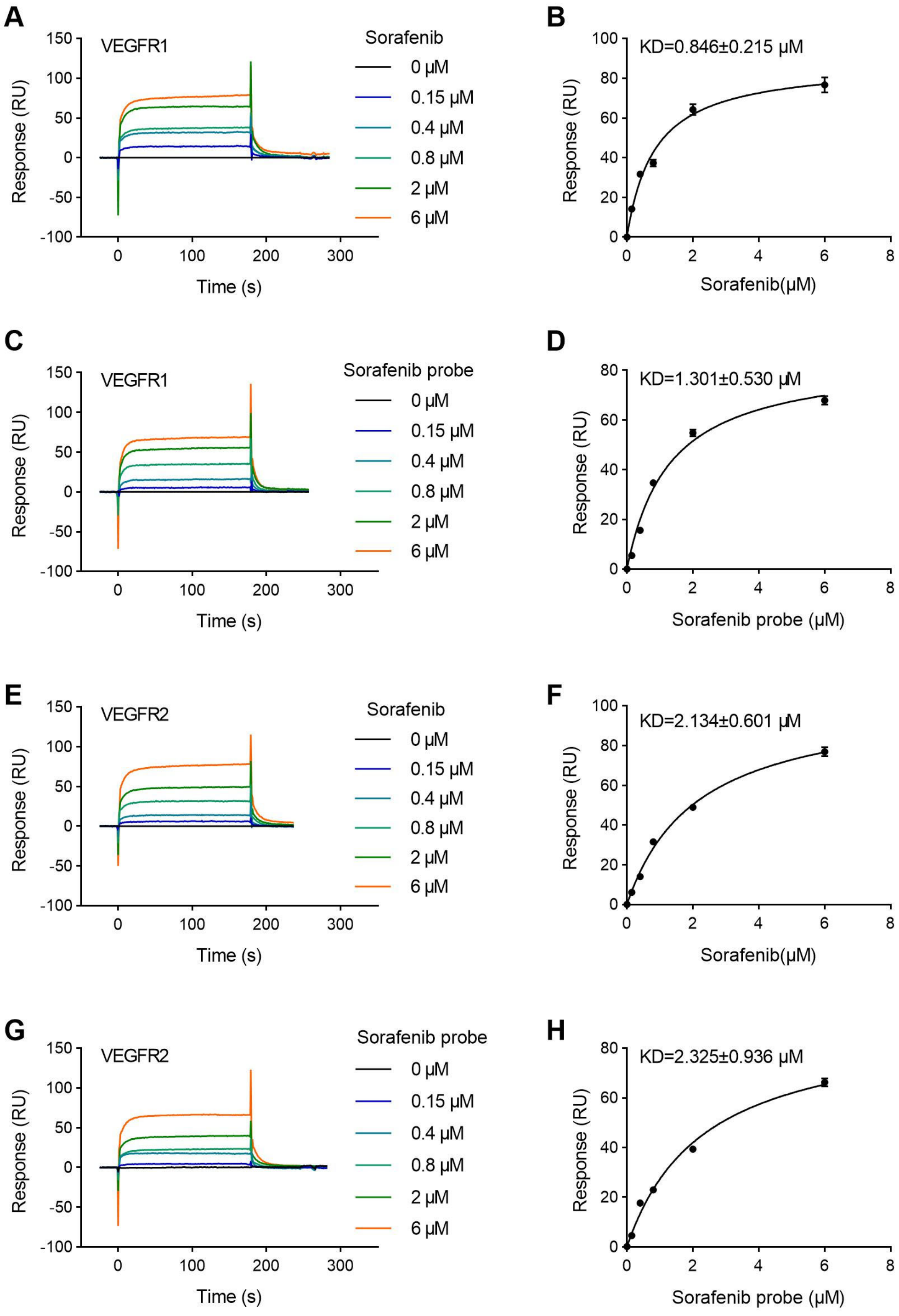

Table S2. Data used to evaluate the stability and reliability of sorafenib probe staining

Fluorescence intensity	Sample	Background
Sample 1	23.580	0.178
Sample 2	23.673	0.036
Sample 3	24.262	0.206
Sample 4	24.928	0.435
Sample 5	25.279	0.053
Sample 6	25.441	0.167
Sample 7	26.486	0.208
Sample 8	27.298	0.503
Sample 9	27.426	0.954
Sample 10	27.630	0.405
Mean(μ)	25.600	0.315
Standard deviation(σ)	1.459	0.260

Table S3. Patient information for sorafenib sensitivity testing

Patient ID	Staining intensity	Days to progression
1	32.602	42
2	55.029	153
3	68.186	93
4	50.994	132
5	55.581	231
6	82.664	426
7	80.545	168
8	51.921	176
9	45.71	53
10	53.129	118
11	32.99	54
12	51.813	71
13	72.895	328
14	26.942	23
15	53.834	127
16	25.387	5
17	49.54	66
18	29.999	16
19	52.68	84
20	29.536	45
21	34.671	75
22	29.051	36
23	71.307	197
24	79.204	254
25	78.397	393
27	74.081	287
28	65.336	204
29	81.675	84
30	78.622	302
31	73.592	225
32	84.837	485
33	79.442	243
34	62.832	189

Fig.S5 Meta-analysis showed that sorafenib had varying effects on different populations

(A) Meta-analysis literature was included in the process. (B) Therapeutic effect of sorafenib versus placebo on OS. (C) Therapeutic effect of sorafenib versus other targeted drugs on OS.

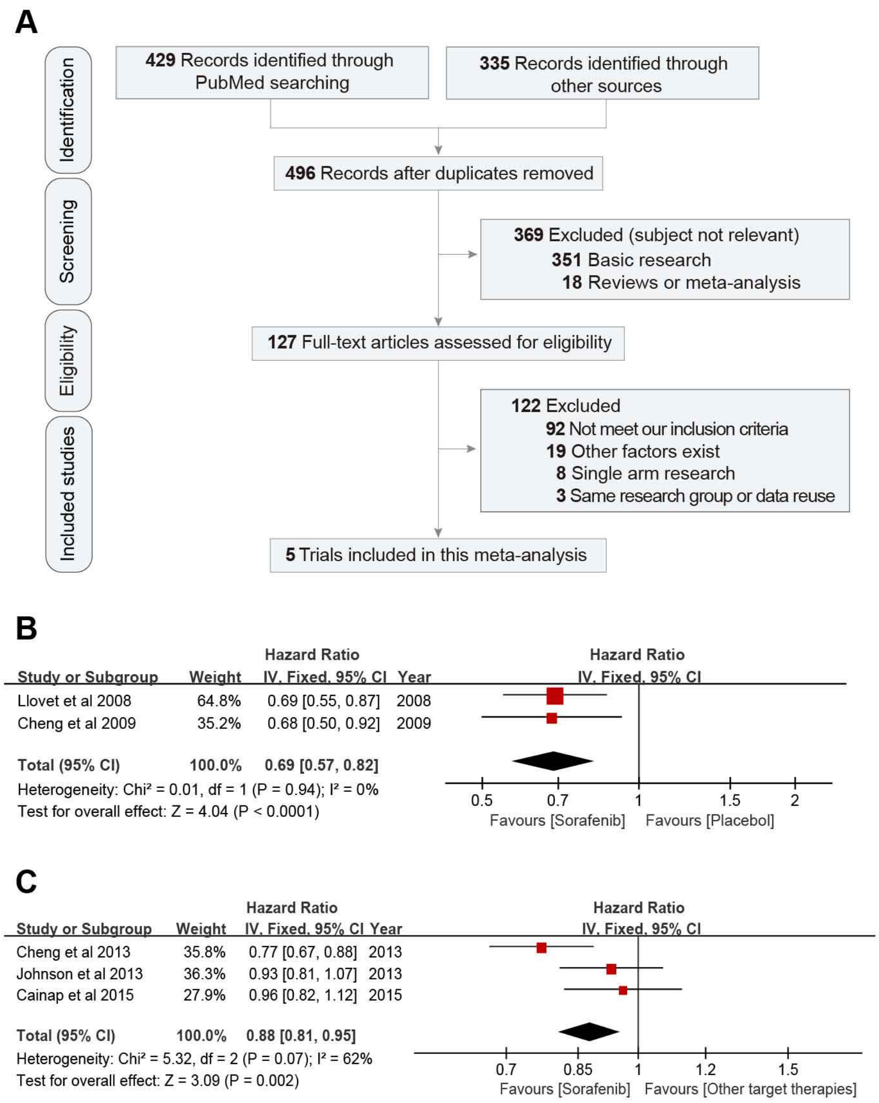

Table S4. Patient Demographics by trial

	Llovet et al.(Llovet et al., 2008) 2008		Cheng et al.(Cheng et al., 2009) 2009		Cheng et al.(Cheng et al., 2013) 2013		Johnson et al.(Johnson et al., 2013) 2013		Cainap et al.(Cainap et al., 2015) 2015	
	sorafenib	placebo	sorafenib	placebo	Sorafenib	sunitinib	sorafenib	brivanib	sorafenib	linifanib
Type	Superiority trial		Superiority trial		Superiority trial/non-inferiority trial		Non-inferiority trial		Superiority trial/non-inferiority trial	
N	299	303	150	76	544	530	578	577	521	514
Median age (years)	65[a]	66[a]	51	52	59	59	60	61	60	59
Male/Female	87/13	87/13	85/15	87/13	84/16	82/18	84/16	84/16	84/16	86/14
ECOG status (0/1/2)	54/38/8	54/39/7	25/69/5	28/67/5	53/47/0	53/47/0	61/39/0	64/36/0	66/34/0	63/37/0
BCLC grade(B/C)	18/82	17/83	5/95	4/96	16/84	13/87	17/78	17/77	20/80	16/84
Child–Pugh grade (A/B+C)	95/5	98/2	97/3	97/3	99/1	100/0	92/8	92/8	95/5	94/6
Cause of disease (HCV/HBV/ Alcohol)	29/11/26	27/9/26	11/71/nr	4/78/nr	22/53/15	21/55/17	21/45/14	20/44/18	25/53/12	25/54/13
Vascular invasion	36	41	36	34	38	43	27	27	41	46
Extrahepatic spread	53	50	69	68	38	36	50	49	57	60
Region (Asia/Other)	0/100	0/100	100/0	100/0	75/25	76/24	64/36	60/40	67/33	66/34
Quality score (Jadad score sheet)	5		5		4		4		5	

a, Data provided in the study are the average age. nr: not reported. ECOG PS, Eastern Cooperative Oncology Group performance status. BCLC, Barcelona Clinic Liver Cancer.

Table S5. Sorafenib versus placebo subgroup analysis on overall survival

Variable	sorafenib versus placebo
ECOG PS 0	**0.70 (0.53, 0.93); p < 0.01**
ECOG PS 1-2	**0.67 (0.53, 0.85); p < 0.01**
No extrahepatic spread	**0.52 (0.39, 0.71); p < 0.01**
Extrahepatic spread	0.84 (0.67, 1.05); p = 0.13
No macroscopic vascular invasion	**0.70 (0.55, 0.90); p < 0.01**
Macroscopic vascular invasion	**0.66 (0.51, 0.87); p < 0.01**
Neither macroscopic vascular invasion nor extrahepatic spread	**0.50 (0.33, 0.77); p < 0.01**
Macroscopic vascular invasion or extrahepatic spread	**0.76 (0.62, 0.93); p < 0.01**
Hepatitis B	0.74 (0.54, 1.03); p = 0.07

Results are expressed as HR and its 95% confidence interval.

Table S6. Sorafenib versus other target therapies (sunitinib/brivanib/linifanib) subgroup analysis on overall survival

**Variable**	**Sorafenib versus sunitinib/brivanib/linifanib**
Asian regions	**0.88 (0.78, 0.99); p = 0.03**
Non-Asian regions	0.81 (0.64, 1.02); p = 0.07
Patients with hepatitis B	0.98 (0.88, 1.10); p = 0.77
Patients with hepatitis C	**0.71 (0.56, 0.89); p < 0.01**
Non-HBV	0.87 (0.76, 1.00); p = 0.06
ECOG PS 0	0.94 (0.83, 1.07); p = 0.35
ECOG PS 1	0.93 (0.79, 1.10); p = 0.40
Macroscopic vascular invasion or extrahepatic spread	0.93 (0.82, 1.05); p = 0.24
Neither Macroscopic vascular invasion nor extrahepatic spread	1.00 (0.72, 1.39); p = 1.00

Results are expressed as HR and its 95% confidence interval.

Figure S6. Relationship between the expression of sorafenib known targets FLT1 and FLT3 and survival, stage or grade in HCC

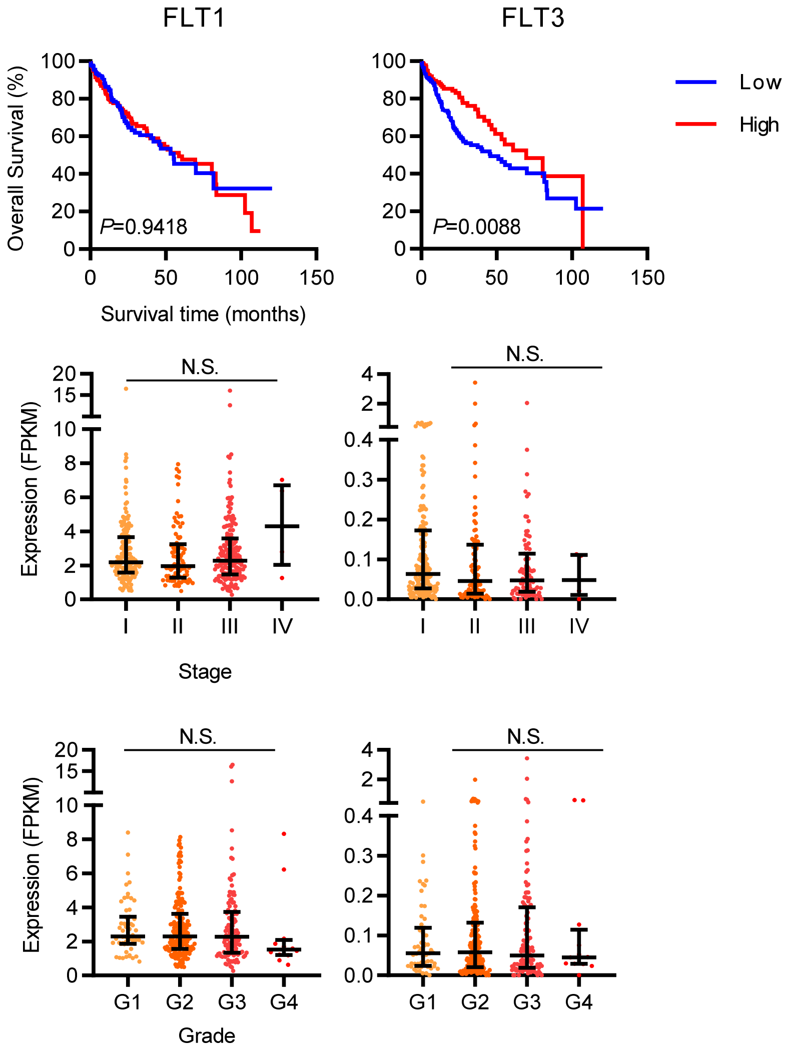

Table S7. Patient information of HCC tissue microarray

Patient ID	Age	Sex	Pathology diagnosis	Grade	Stage
1	39	M	Hepatocellular carcinoma	II-III	II
2	33	M	Hepatocellular carcinoma	II-III	II
3	47	M	Hepatocellular carcinoma	III	II
4	65	M	Hepatocellular carcinoma	III	II
5	52	M	Hepatocellular carcinoma	II	II
6	46	F	Hepatocellular carcinoma	III	IIIB
7	52	F	Hepatocellular carcinoma	III	I
8	47	M	Hepatocellular carcinoma	II	IIIA
9	41	M	Hepatocellular carcinoma	II	II
10	36	M	Hepatocellular carcinoma	II	II
11	61	M	Hepatocellular carcinoma	II	II
12	60	M	Hepatocellular carcinoma	II	II
13	52	M	Hepatocellular carcinoma	II	IIIA
14	44	M	Hepatocellular carcinoma	II	II
15	73	F	Hepatocellular carcinoma	II	IIIA
16	60	F	Hepatocellular carcinoma	I-II	II
17	18	M	Hepatocellular carcinoma	II	II
18	67	M	Hepatocellular carcinoma	II	II
19	43	M	Hepatocellular carcinoma	I	II
20	75	M	Hepatocellular carcinoma	I	IIIA
21	32	M	Hepatocellular carcinoma	II	II
22	50	F	Hepatocellular carcinoma	II	IIIB
23	57	M	Hepatocellular carcinoma	I-II	II
24	43	M	Hepatocellular carcinoma	I-II	IIIA
25	50	M	Hepatocellular carcinoma	I-II	IVA
26	42	M	Hepatocellular carcinoma	II	II
27	53	M	Hepatocellular carcinoma	II	II
28	40	M	Hepatocellular carcinoma	I-II	IIIA

29	55	M	Hepatocellular carcinoma	III	IIIA
30	42	F	Hepatocellular carcinoma	II	II
31	38	M	Hepatocellular carcinoma	II	II
32	40	M	Hepatocellular carcinoma	III	IIIA
33	55	M	Hepatocellular carcinoma	III	II
34	46	F	Hepatocellular carcinoma	II	II
35	47	M	Hepatocellular carcinoma	II	II
36	51	M	Hepatocellular carcinoma	II	IIIA
37	38	M	Hepatocellular carcinoma	III	IIIA
38	50	M	Hepatocellular carcinoma	II	II
39	43	M	Hepatocellular carcinoma	III	IIIA
40	58	M	Hepatocellular carcinoma	I-II	II
41	31	M	Hepatocellular carcinoma	I-II	II
42	44	M	Hepatocellular carcinoma	I	II
43	52	M	Hepatocellular carcinoma	II	II
44	48	M	Hepatocellular carcinoma	II	II
45	73	M	Hepatocellular carcinoma	III	II
46	50	F	Hepatocellular carcinoma	I	II
47	69	F	Hepatocellular carcinoma	II	II
48	66	M	Hepatocellular carcinoma	I-II	IIIA
49	53	M	Hepatocellular carcinoma	I	II
50	32	M	Hepatocellular carcinoma	II	II
51	62	M	Hepatocellular carcinoma	I	II
52	59	M	Hepatocellular carcinoma	II	II
53	79	F	Hepatocellular carcinoma	III	II
54	74	M	Hepatocellular carcinoma	II-III	IIIA
55	50	M	Hepatocellular carcinoma	II	II
56	67	M	Hepatocellular carcinoma	II	II
57	50	F	Hepatocellular carcinoma	I-II	IIIB
58	62	M	Hepatocellular carcinoma	I-II	IIIA

59	50	M	Hepatocellular carcinoma	III	IIIA
60	33	M	Hepatocellular carcinoma	II	IIIA
61	37	M	Hepatocellular carcinoma	I	IIIA
62	30	M	Hepatocellular carcinoma	II	IIIA
63	60	M	Hepatocellular carcinoma	III	IIIA
64	28	F	Hepatocellular carcinoma	II	IIIB
65	56	F	Hepatocellular carcinoma	I-II	IIIA
66	54	M	Hepatocellular carcinoma	III	IIIA
67	63	M	Hepatocellular carcinoma	II	IIIA
68	37	M	Hepatocellular carcinoma	III	II
69	61	M	Hepatocellular carcinoma	II	IIIA
70	35	M	Hepatocellular carcinoma	III	IIIA
71	17	F	Hepatocellular carcinoma	II	IIIA
72	35	M	Hepatocellular carcinoma	III	IIIA
73	48	M	Hepatocellular carcinoma	III	IIIA
74	72	M	Hepatocellular carcinoma	II	IIIA
75	58	M	Hepatocellular carcinoma	II	IIIA

Figure S7. Sorafenib probe staining intensity was not significantly different from gender and age

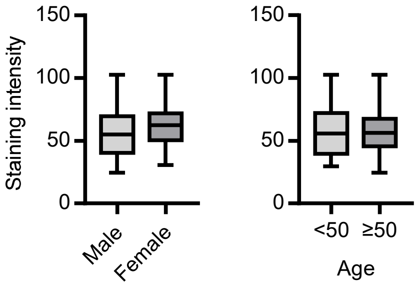

Figure S8. Representative images of sorafenib staining on HCC tissue microarray

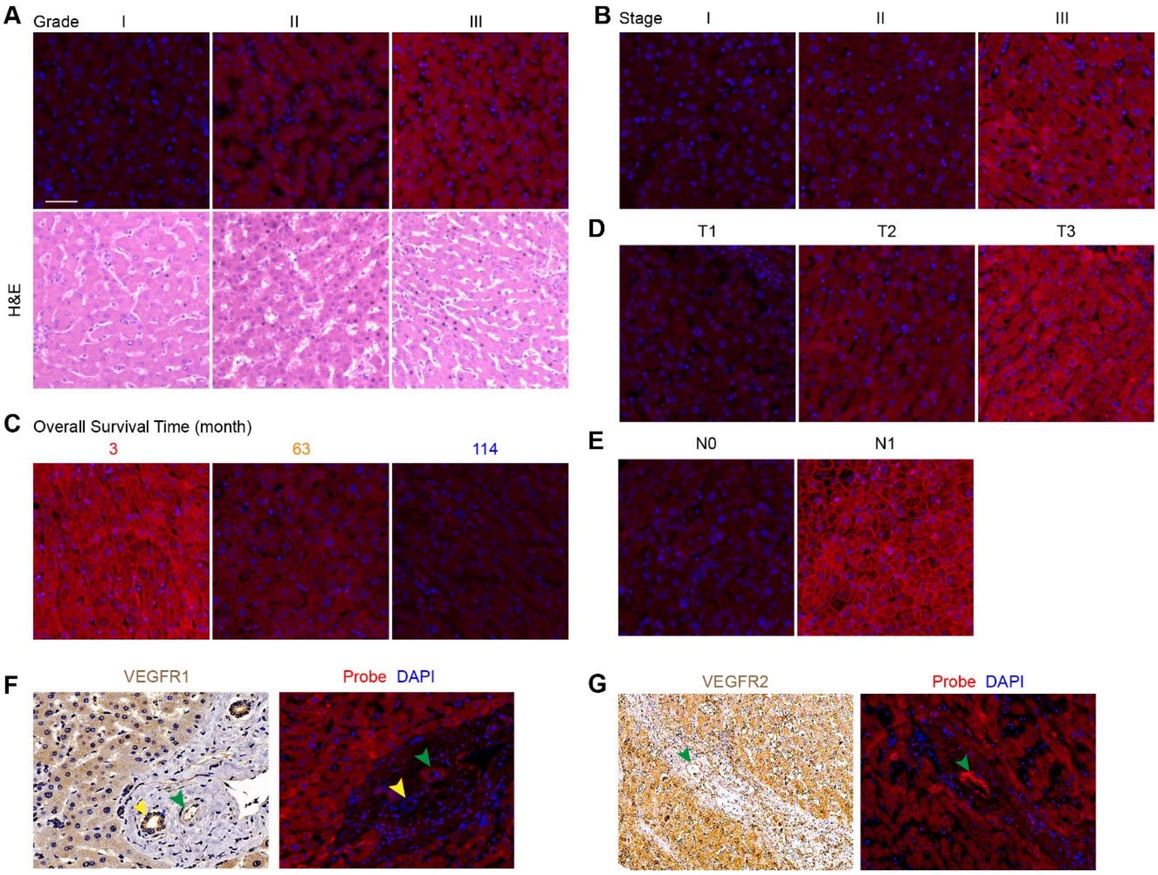

Figure S9. Different Sequence of probe staining and immunofluorescence staining

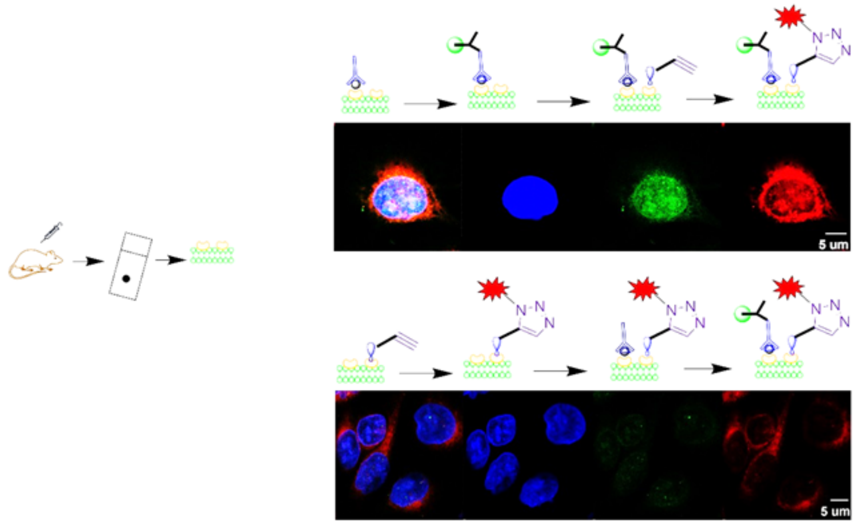

Transparent Methods

Materials

The following materials were used in this study: imatinib and sorafenib (MeilunBio, Dalian, China); CCK-8 kit (Beyotime Biotechnology, Shanghai, China); immunofluorescence staining kit with FITC-labeled goat anti-rabbit IgG and cell lines (KeyGEN BioTECH, Nanjing, China); antibodies to CD117, VEGFR2, PDGFR-β, p-VEGFR2, p-PDGFR-β (Affinity Bioreagents, Colorado, USA); Anti-Hepatocyte Specific Antigen antibody (Abcam).

Cell culture

GIST882, MHCC97H, MHCC97L, HepG2, Huh7 and PLC-PRF-5 cells were cultured in DMEM (HyClone), SNU-182, SNU-387, SNU-398, SNU-423, and SNU-449 cells were cultured in RPMI-1640 (HyClone) supplemented with 10% (v/v) fetal bovine serum (Thermo Fisher Scientific) at 37 °C in humidified atmosphere containing 5% CO_2. The cell lines were tested to determine the presence of *Mycoplasma* before use. Cell identification was provided by KeyGen Biotech (Nanjing, China).

Cell proliferation assay

CCK-8 assay determines the cell proliferation. PLC-PRF-5, MHCC-97L, and MHCC-97H (5 × 10^3 cells/mL) were seeded in 96-well culture plates (n = 3). After overnight incubation, the cells were treated with various concentrations of N-desmethyl imatinib, N-desmethyl imatinib probe, sorafenib, or sorafenib probe. After 48 h incubation, cell viability was measured after the addition of 10 μL CCK-8 solution at 37 °C for 2 h. Optical density was determined at 450 nm with a microplate reader (Multiskan™ FC, Thermo Scientific, Waltham, MA, USA). The ratio of absorbance to the control group was analyzed by nonlinear regression analysis using GraphPad Prism version 7 for Windows to obtain the IC_{50} value.

Synthesis of sorafenib probe

Under dark conditions, $[Pd(cinnamyl)Cl]_2$ (1.6 mg, 0.5 mol%), RockPhos (8.4 mg, 1.5 mol%), Cs_2CO_3 (391 mg, 1.2 mmol), and toluene (5 mL) were added to a glass vial that was sealed with a screw cap fitted with a PTFE/silicone septum and removed from the glovebox. Sorafenib (279 mg, 0.60 mmol) and alcohol (100 mg, 0.72 mmol) in 1 mL of toluene were then added using a microsyringe. The vial was then placed on a temperature-controlled aluminum plate set to 90 °C, and the solution was stirred for 16 h. Afterward, the vial was removed from the heating block and cooled to room temperature. An aliquot was filtered through a small plug of silica that was then washed with dichloromethane, and the solvent was evaporated under reduced pressure. The crude product was subjected to macroporous resin column chromatography (methanol/water) to produce the probe (125.8 mg, 37%).

Patient samples and ethics

We complied with all relevant ethical regulations. We collected more than 300 paraffin-embedded specimens of patients with HCC and analyzed the clinical data integrity. A total of 33 patients took sorafenib after they underwent surgery. Their paraffin specimens were used for sorafenib probe staining detection. The staining results were further conducted correlation analysis with clinical data. These

cases were from Tianjin Medical University General Hospital, Tianjin Cancer Hospital, Beijing Shunyi Hospital, Shandong Shouguang Hospital, Tangshan Coal Hospital, Shanxi Houma People’s Hospital, 541st Hospital of the Chinese People's Liberation Army, Liaoning Provincial People's Hospital and Huashan Hospital Affiliated to Fudan University.

Probe staining assay for cells

A 24-well plate with a climbing film was plated with about 7×10^4 log phase cells, and 500 μL of the complete medium was added to each well. After 9 h, the probe was added (final concentration: 10 μM), and an equal amount of DMSO was added to the control group. After incubation for 8 h, the medium was discarded, PBS was immersed thrice for 3 min, and 200 μL of cold methanol was added for 20 min at room temperature. Thereafter, methanol was discarded, and PBS was immersed thrice for 3 min and irradiated with ultraviolet light (365 nm) for 1 h. The cells were incubated with 0.1% TritonX-100 for 10 min, washed with PBS thrice for 3 min, added with 5% FBS in PBS solution, and blocked at room temperature for 30 min. The cells were washed thrice with PBS and added with a clicking reaction solution for 1 h, but the solution was protected from light exposure. The cells were washed four times with PBS until they became colorless. Finally, the cells were stained with DAPI and photographed using a laser confocal microscope (Leica, Germany) under the same acquisition condition. Images of the probe fluorescent staining channels were analyzed with ImageJ and the mean fluorescence intensity of each sample was calculated.

Probe staining assay for sections

Paraffin sections were immersed in xylene for 15 min, dewaxed with xylene II for 15 min, placed in 100% alcohol for 5 min, 100% alcohol II for 5 min, 95% alcohol for 5 min, 80% alcohol for 5 min, tap water for 5 min, rinsed with distilled water for 3 min, and washed with PBS thrice for 3 min for rehydration. The tissue sections were then placed in a 50-fold diluted EDTA (pH 8) repair solution, preheated in a microwave defrost mode for 6 min until it slightly boiled, and maintained at low and medium heat for 15 min. Afterward, the cells were naturally cooled for 20–30 min for antigen retrieval and then immersed in PBS thrice for 3 min. The PBS outside the specimen was wiped off with a filter paper and placed in a humid chamber. Subsequently, 0.3% Triton X-100 was added dropwise and incubated for 10 min at room temperature. The cells were immersed in PBS thrice for 3 min at each time, after which 2% BSA was added for 30 min. The tissue was incubated with probe solution (10 μM) for 1 h at 37 °C, and the control group was added with the same amount of PBS. The tissue was immersed in PBS thrice for 3 min. PBS was added to cover the tissue, which was exposed to UV light (365 nm) for 1 h. Thereafter, the working solution of the click solution was added for the click reaction for 2 h and immersed in PBS four times for 3 min at each time, washed until the samples became colorless, and sealed with DAPI. The samples were photographed with a laser confocal microscope or a slice scanner under the same acquisition condition. Images of the probe fluorescent staining channels were analyzed with ImageJ and the mean fluorescence intensity of each sample was calculated.

Catching targets using the probe

The log phase cells were incubated with the probe (final concentration 10 μM) for 8 h, and the medium was discarded. A total of 400 μL of RIPA strong lysate was added to each dish and allowed to stand for

30 min on ice. The cells were scraped and transferred to an EP tube. The samples were centrifuged at 12,000 rpm for 10 min at 4 °C. The supernatant was obtained, and the click reaction solution was added for 1 h. The small molecule was treated with a 5 kD ultrafiltration tube and 30 μL of streptavidin magnetic beads. The mixture was incubated overnight at 4 °C with gentle rotation. The cells were washed four times with RIPA weak lysate, stained with Coomassie blue via SDS-PAGE, and identified through mass spectrometry after in-gel digestion was conducted.

Molecular docking

KIT (ID 1PKG), BRAF (ID 4E26), RAF1 (ID 3OMV), VEGFR1 (ID 3HNG), and VEGFR2 (ID 2XIR) in PDB file format were downloaded from the Protein Data Bank(Berman, 2000). Imatinib and sorafenib in SDF file format were downloaded from PubChem(Kim et al., 2016). Molecular docking was performed using Schrödinger Maestro 8.0. The ligand and protein complex were saved in PDB file format and further analyzed with Pymol 2.2.

Surface plasmon resonance (SPR) assay

SPR experiments were performed using a Biacore 3000 instrument (GE Healthcare, Piscataway, NJ, USA) at 25°C. CD117 was purchased from Novus Biologicals, and VEGFR1 and VEGFR2 were purchased from Sigma-Aldrich. CD117, VEGFR1, and VEGFR2 were immobilized on CM5 sensor chips using the Biacore Amine Coupling Kit in accordance with the manufacturer's instructions. Briefly, the chip was activated using a 1:1 mixture of 0.2 M N-ethyl-N'-(3-dimethylaminopropyl) carbodiimide and 0.05 M N-hydroxysuccimide at 10 μL/min for 7 min. CD117, VEGFR1, and VEGFR2 were coated on the chip at 150 μg/mL in 10 mM sodium acetate buffer (pH 5.0) for 2.5, 3.5, 3 min at 10 μL/min to a level of 8700, 9200, 9800 response units. The unoccupied binding sites were blocked using 1 M ethanolamine at pH 8.5. N-desmethyl imatinib, N-desmethyl imatinib probe, sorafenib and sorafenib probes were dissolved to 10 mM by 100% DMSO and diluted to 10 μM by 1×PBS (pH 7.4). Finally, a series of analytes (N-desmethyl imatinib and N-desmethyl imatinib ranging from 0.035–2.0 μM, sorafenib and sorafenib probe ranging from 0.15-6.0 μM) was used as kinetic analytes. (Theoretical Rmax: Imatinib-CD117 38.36 RU, Imatinib probe-CD117 30.67 RU, sorafenib-VEGFR1 84.51 RU, sorafenib probe-VEGFR1 103.01 RU, sorafenib-VEGFR2 36.86 RU, sorafenib probe-VEGFR2 44.93 RU.) 1×PBS (pH 7.4, 0.1% DMSO) was used as the running buffer for analyte dilution. All buffers were filtered and degassed prior to use. For kinetic analysis, a blank cell without protein was used as the reference cell and performed other operations as mentioned before, and the data were analyzed using the BIA evaluation software (Version 4.1).

Immunofluorescence staining

The cells were fixed with 4% formaldehyde in phosphate-buffered saline (PBS) for 5 min, permeabilized with 0.2% Triton X-100, blocked with 3% bovine serum albumin, and incubated overnight with primary antibodies at 4 °C. FITC-labeled secondary antibodies were incubated for 1 h at room temperature. Each step was followed by two washing procedures with for 5 min. Finally, the cells stained with 4′,6-diamidino-2-phenylindole (DAPI; Sigma, USA) were mounted and viewed using a laser scanning confocal microscope A1 (Nikon, Japan). The sequence of probe staining and immunofluorescence staining was tested. (Fig. S7)

Preparation of tissue microarrays

GIST tissue microarrays containing 24 cases and HCC tissue microarrays consisting of 75 cases were purchased from the US Biomax for IHC and probe staining. Tissue blocks of the tissue array were collected within 5 years. Each single tissue spot on every array slide was individually examined by certified pathologists in accordance with the published standardizations of the World Health Organization for diagnosis, classification, and pathological grade. The hospital and the patients consented to include the specimens in the study. All tissues were collected under the highest ethical standards. Each donor was completely informed and provided consent. All human tissues were collected under Health Insurance Portability and Accountability Act (HIPPA)-approved protocols. Probe staining and immunohistochemical staining were performed on the tissue microarrays with the same sample distribution, and results of the same sample were continuously observed.

Uniformity and signal variability assessment of the probe staining assay

To evaluate the stability and reliability of the probe staining system, we analyzed the staining performance of the probe on the basis of the signal-to-background ratio (S/B), signal-to-noise ratio (S/N), coefficient of variation (CV) and Z-factor (Z') by using the following formula: (Zhang et al., 1999)

$$S/B=\frac{\mu_s}{\mu_b}$$

$$S/N=\frac{\mu_s-\mu_b}{\sigma_b}$$

$$CV=\frac{\sigma}{\mu}\times 100\%$$

$$Z'=1-\frac{3\times(\sigma_s+\sigma_b)}{\mu_s-\mu_b}$$

where μ, σ, s, and b represent the mean, standard deviations, signal, and background, respectively.

Super-resolution (STORM) imaging

The colocalization of the imatinib probe with CD117 was observed with Nikon Super-Resolution Microscope N-STORM. Immediately before imaging was conducted, the samples were covered with an imaging medium freshly prepared in accordance with the manufacturer's instructions (Dudok et al., 2015), containing 5% (m/v) glucose, 0.1 M mercaptoethylamine, 1 mg mL^{-1} glucose oxidase and catalase (2.5 μL mL^{-1} of aqueous solution from Sigma, approximately 1,500 U mL^{-1} final concentration) in Dulbecco's PBS (Sigma). Finally, coverslips were sealed with nail polish, and the slides were transferred to the microscope setup after 10 min. STORM imaging was performed for up to 3 h after the specimens were covered. STORM images were processed using the N-STORM module in NIS-Elements AR.

Western blot analysis

Cells were washed with PBS and lysed in ice-cold lysis buffer with protease Inhibitor Cocktail (Sigma) on ice for 30 min. Lysates were separated through electrophoresis and transferred onto polyvinylidene difluoride membranes (Millipore). The membranes were blocked, incubated with primary antibody against CD117 (1:1,000, Affinity), VEGFR2 (1:1,000, Affinity), PDGFR-β (1:1,000, Affinity), p-VEGFR2 (1:1,000, Affinity), and p-PDGFR-β (1:1,000, Affinity) at 4 °C overnight, and incubated with a horseradish

peroxidase-conjugated goat anti-rabbit IgG secondary antibody (Thermo Scientific) for 1 h at room temperature. Protein expression was assessed using an enhanced chemiluminescent substrate (Millipore) and exposed to a chemiluminescent film.

Cell-derived xenograft model

Animal experiments were conducted in accordance with the National Institutes of Health Animal Use Guidelines. All of the experimental protocols were approved by the Institutional Animal Care and Use Committee at Tianjin International Joint Academy of Biomedicine. A total of 1×10^7 cells were injected subcutaneously to nude mice. When tumor volume reached approximately 50 mm^3, the mice were treated with 30 mg/kg sorafenib or the vehicle. The diameters of the tumors were serially measured with a digital caliper every 3 days, and their volumes were calculated using the following formula: volume = $(length \times width^2)/2$. The mice were euthanized 25 days after cell injection. The tumors were sectioned into paraffin sections and then stained and analyzed by probe staining assay for sections. The tumor inhibition rate was calculated by the following formula: tumor inhibition rate = 1 − mean tumor volume (sorafenib) / mean tumor volume (control).

Bioinformatics analysis

The clinical information and gene expression data of liver hepatocellular carcinoma in The Cancer Genome Atlas (TCGA) were downloaded using the R package TCGAbiolinks(Colaprico et al., 2016) and analyzed via GraphPad Prism version 7.00. Principal component analysis (PCA) was performed using SIMCA 14.1. COX regression analysis was conducted with SPSS 21.0. Gene set enrichment analysis (GSEA) was carried out using R package clusterProfiler(Yu et al., 2012).

Statistical analysis

Statistical analyses were performed using GraphPad Prism version 8 for Windows and R 3.5.1. Statistically significant differences were calculated using Student's *t*-test, one-way ANOVA, Pearson's correlation, and Kaplan–Meier as needed. $P < 0.05$ was considered significant.

References

Berman, H.M. (2000). The Protein Data Bank. Nucleic Acids Research *28*, 235-242.

Cainap, C., Qin, S., Huang, W.T., Chung, I.J., Pan, H., Cheng, Y., Kudo, M., Kang, Y.K., Chen, P.J., Toh, H.C.*, et al.* (2015). Linifanib versus Sorafenib in patients with advanced hepatocellular carcinoma: results of a randomized phase III trial. J Clin Oncol *33*, 172-179.

Cheng, A.L., Kang, Y.K., Chen, Z., Tsao, C.J., Qin, S., Kim, J.S., Luo, R., Feng, J., Ye, S., Yang, T.S.*, et al.* (2009). Efficacy and safety of sorafenib in patients in the Asia-Pacific region with advanced hepatocellular carcinoma: a phase III randomised, double-blind, placebo-controlled trial. Lancet Oncol *10*, 25-34.

Cheng, A.L., Kang, Y.K., Lin, D.Y., Park, J.W., Kudo, M., Qin, S., Chung, H.C., Song, X., Xu, J., Poggi, G.*, et al.* (2013). Sunitinib versus sorafenib in advanced hepatocellular cancer: results of a randomized phase III trial. J Clin Oncol *31*, 4067-4075.

Colaprico, A., Silva, T.C., Olsen, C., Garofano, L., Cava, C., Garolini, D., Sabedot, T.S., Malta, T.M., Pagnotta, S.M., Castiglioni, I.*, et al.* (2016). TCGAbiolinks: an R/Bioconductor package for integrative analysis of TCGA data. Nucleic Acids Res *44*, e71.

Dudok, B., Barna, L., Ledri, M., Szabo, S.I., Szabadits, E., Pinter, B., Woodhams, S.G., Henstridge, C.M., Balla, G.Y., Nyilas, R.*, et al.* (2015). Cell-specific STORM super-resolution imaging reveals nanoscale organization of cannabinoid signaling. Nat Neurosci *18*, 75-86.

Johnson, P.J., Qin, S., Park, J.W., Poon, R.T., Raoul, J.L., Philip, P.A., Hsu, C.H., Hu, T.H., Heo, J., Xu, J.*, et al.* (2013). Brivanib versus sorafenib as first-line therapy in patients with unresectable, advanced hepatocellular carcinoma: results from the randomized phase III BRISK-FL study. J Clin Oncol *31*, 3517-3524.

Kim, S., Thiessen, P.A., Bolton, E.E., Chen, J., Fu, G., Gindulyte, A., Han, L., He, J., He, S., Shoemaker, B.A.*, et al.* (2016). PubChem Substance and Compound databases. Nucleic Acids Res *44*, D1202-1213.

Llovet, J.M., Ricci, S., Mazzaferro, V., Hilgard, P., Gane, E., Blanc, J.F., de Oliveira, A.C., Santoro, A., Raoul, J.L., Forner, A.*, et al.* (2008). Sorafenib in advanced hepatocellular carcinoma. N Engl J Med *359*, 378-390.

Yu, G., Wang, L.G., Han, Y., and He, Q.Y. (2012). clusterProfiler: an R package for comparing biological themes among gene clusters. OMICS *16*, 284-287.

Zhang, J.H., Chung, T.D., and Oldenburg, K.R. (1999). A Simple Statistical Parameter for Use in Evaluation and Validation of High Throughput Screening Assays. J Biomol Screen *4*, 67-73.
